# Sex Representation and User Preferences in Pain Drawing Body Charts in Back Pain Research: Multimethod Study

**DOI:** 10.2196/76175

**Published:** 2026-02-09

**Authors:** Sonja Schläpfer, Casey Murphy, Yanick X Lukic, Concepción Campos-Asensio, Giovanni Colacicco, Beatrice A Lauber, Sophie Masneuf, Luana Nyirö, Georg W Kajdi, Reto Sutter, Christoph J Laux, Philipp Ackermann, Jesús López-Alcalde, Claudia M Witt

**Affiliations:** 1 Complementary and Integrative Digital Health, Institute of Primary Care, University of Zurich and University Hospital Zurich, Sonneggstrasse 6, Zurich, 8091, Switzerland, 41 44 255 94 51; 2 Institute of Computer Science, School of Engineering, Zurich University of Applied Sciences, Winterthur, Switzerland; 3 School of Medicine, University of St.Gallen, St.Gallen, Switzerland; 4 Medical Library, Hospital Universitario de Getafe, Madrid, Spain; 5 Institute of Anatomy, University of Zurich, Zurich, Switzerland; 6 Neuroscience Center Zurich, ETH and University of Zurich, Zurich, Switzerland; 7 Department of Chiropractic Medicine, Balgrist University Hospital, University of Zurich, Zurich, Switzerland; 8 Radiology Department, Balgrist University Hospital, University of Zurich, Zurich, Switzerland; 9 University Spine Center Zurich, Balgrist University Hospital, University of Zurich, Zurich, Switzerland; 10 Faculty of Medicine, Universidad Francisco de Vitoria (UFV), Madrid, Spain; 11 Instituto Ramón y Cajal de Investigación Sanitaria (IRYCIS), Unidad de bioestadística clínica, Hospital Universitario Ramón y Cajal, (CIBERESP), Cochrane Madrid, Madrid, Spain

**Keywords:** pain drawing, pain map, body chart, sex, gender, sex/gender bias, back pain

## Abstract

**Background:**

Pain drawing (PD) body charts are widely used in back pain research, but the representation of sex in these charts has not been systematically evaluated.

**Objective:**

This study aims to evaluate sex representation in PD body charts used in back pain research, assess the perception of a newly designed sex-neutral body chart, and explore user preferences for sex representation in PD body charts.

**Methods:**

We conducted a multimethod study comprising: (1) a scoping review to assess sex representation and the reporting of sex in PD body charts in back pain literature, (2) an expert opinion study where anatomy experts evaluated the perceived sex of extracted body charts, and (3) a survey among a representative sample of UK adults with and without back pain to assess the perception of a newly designed sex-neutral body chart and explore preferences for sex representation in PD body charts.

**Results:**

From 349 full-text papers, 108 articles met the inclusion criteria. Most (103/108, 95.4%) did not report the sex of the body charts used, and only 5.6% (6/108) included both male and female charts. Experts showed fair to moderate agreement (Fleiss κ=0.306; Gwet AC1=0.456) in assessing the sex of charts, with most charts assessed as male based on majority ratings (59/108, 54.6%) and classified as male-biased relative to the sex distribution of study participants (76/108, 67.7%). The newly designed sex-neutral body chart was perceived as sex-neutral by 68.5% (204/298) of survey participants across diverse groups. However, perceptions varied by racial group: 73% (181/248) of White participants viewed it as sex-neutral, compared to 42.5% (17/40) of participants from smaller racial groups (*χ*²_2_=15.9; *P*=.001). Female participants slightly preferred female charts (85/154, 55.2%); male participants preferred sex-neutral ones (88/144, 61.1%). Nonetheless, most female participants (82/154, 53.3%) and male participants (107/144, 74.3%) considered the option to choose between male, female, and sex-neutral chart versions unimportant.

**Conclusions:**

Our study reveals reporting gaps and a predominant male bias in the representation of sex in PD body charts used in back pain research. The newly developed sex-neutral body chart was widely perceived as sex-neutral, offering a promising step toward more inclusive pain assessment. However, variations in perception across racial groups highlight the need for cultural considerations in design. These findings underscore the potential of sex-neutral and culturally sensitive body charts to enhance the inclusivity and equity of back pain research and clinical practice.

## Introduction

A pain drawing (PD) is an illustration of the human body that allows individuals to draw the location and extent of their pain. These visual tools support the assessment of subjective pain, may help determine appropriate treatment strategies, and facilitate communication between patients and health care professionals [1]. Since their introduction in 1949, PDs have evolved from traditional paper-and-pencil formats into digital and, in some cases, 3D representations [2]. The body charts used in these PDs vary considerably [2], with some representing male or female figures, while others may be perceived as sex-neutral.

The first sex-specific body charts, featuring both male and female versions, were introduced in 1988 by Uden et al [3], marking an important advancement in acknowledging the need for diversity in pain reporting tools [2]. However, despite this progress, subsequent research has largely neglected the role of diversity in body chart design. Most studies have examined how a participant’s sex influences pain depiction by comparing PDs from female and male participants [4,5], while overlooking whether the sex of the body chart itself influences pain reporting. In addition to this gap, it remains unclear to what extent male, female, and sex-neutral body charts are currently used in pain research.

The sex of body charts used in pain research is often poorly documented, and the prevalence of different sex representations remains largely unknown. Preliminary studies suggest that male or sex-neutral charts with male features are more commonly used than female or truly sex-neutral alternatives. Most body charts are described using terms such as “human body outline,” without explicitly identifying them as male, female, or sex-neutral [6]. This ambiguity raises important questions about how a “universal” human body is visually represented across sexes and genders, and how individuals perceive and interpret these depictions. A prevailing assumption among researchers is that most body charts are sex-neutral with distinctly male features, while truly sex-neutral representations are rare [1,7]. However, this perspective is not grounded in a comprehensive review, highlighting the need for further investigation into potential sex and gender biases in body chart design.

Another important consideration is whether individuals prefer male, female, or sex-neutral body charts. While some studies indicate a preference for sex-specific body charts [1,8,9], their findings may be limited by the fact that body charts intended to be sex-neutral are not always perceived or interpreted as such. Understanding individuals’ preferences is critical, as these preferences may influence how users engage with the charts, their comfort in using them, and the accuracy of their pain reporting. When individuals feel represented by the body chart, they may be more likely to provide accurate and detailed pain descriptions, potentially leading to improved diagnostic outcomes [1]. Exploring these preferences also supports the development of inclusive, user-centered tools that reflect the diversity of patient populations—an essential step toward more equitable health care.

This study examines how sex is represented in PD body charts within the back pain literature, analyzing both the descriptions provided in published articles and anatomy experts’ assessments of the perceived sex of the charts. Furthermore, we evaluated a newly developed sex-neutral body chart, designed by an interdisciplinary team of illustrators, computer scientists, physicians (specializing in anatomy, orthopedics, radiology, and integrative medicine), and psychologists [10]. Our aim was to determine whether this chart is perceived by individuals as truly sex-neutral, in alignment with its intended design. We also explored a range of individual factors that may influence these perceptions, alongside participants’ preferences for sex-specific versus sex-neutral body charts. Taken together, this study sought to address the following research questions:

1. What is the prevalence of male, female, and sex-neutral PD body charts in the back pain literature—from its inception to the present—as reported in the articles and as assessed by experts? Additionally, what is the level of agreement among experts in their sex-based classifications of the charts?
2. How do individuals perceive a body chart explicitly designed to be sex-neutral, and what individual factors influence whether they interpret the chart as male, female, or sex-neutral?
3. In the context of back pain assessment, do individuals prefer male, female, or sex-neutral body charts, and how important is having a choice to them?

Throughout this paper, we use the terms *sex* and *gender* in accordance with the SAGER (Sex and Gender Equity in Research) guidelines [11]. We define *sex* in body charts based on anatomical characteristics, including primary and secondary sexual organs typically associated with male and female bodies, and in humans as sex assigned at birth. *Gender* refers to socially constructed roles, behaviors, and identities in humans.

## Methods

### Study Design

This research adopted a multimethod design comprising a scoping review, an expert opinion study, and a survey—each focusing on different aspects of sex representation in PD body charts within the context of back pain. The expert opinion study incorporated insights from anatomy experts across various medical and health sciences disciplines, while the survey gathered responses from individuals both with and without back pain. This inclusive approach ensured the integration of diverse perspectives throughout the study. We submitted the study synopsis to the Ethics Committee of Zurich, Switzerland, and after review, they stated that the study does not fall under the regulation of the Human Research Act of Switzerland (ethics ID: 2022‐01475). The scoping review protocol was registered in the Open Science Framework on July 1, 2024, prior to starting the process of selecting studies for the review [12].

### Ethical Considerations

The study was conducted in accordance with ethical standards. Privacy and confidentiality were ensured by pseudonymizing all data in the expert opinion study and by collecting anonymized data in the survey, with no personal identifiers recorded. All data were securely stored. Participation was voluntary, and participants were informed of their right to withdraw at any time. Participants provided electronic informed consent. Each participant received £0.75 (US $1) as compensation for their time.

### Inclusion Criteria

#### Review

The scoping review considered studies that have used, designed, or evaluated PDs with a full-body or torso template in paper or digital format. Studies were included if they assessed pain location and involved adults (aged ≥18 y) with back pain, regardless of participants’ demographic characteristics (such as age, sex, or pain type and severity). The review considered all primary research studies published in English or German, conducted in any country or clinical setting. Secondary studies—including systematic reviews, narrative reviews, clinical practice guidelines, and consensus documents—were excluded.

#### Expert Opinion Study

The expert opinion study included experts with academic qualifications and professional expertise in medicine or related health sciences, with an equal distribution of male and female experts.

#### Survey

The survey included English-speaking adults aged 18 years or older who resided in the United Kingdom, had provided informed consent, and either had or had not experienced back pain, regardless of its type or severity. To ensure that the sample was approximately representative of the UK population, participants also had to meet demographic criteria for sex, age, and race based on simplified UK Census data.

### Recruitment Procedure

#### Review

The search strategy for the scoping review aimed to identify relevant published studies. An initial, limited search of PubMed was conducted to locate key articles, followed by an analysis of the text words in titles and abstracts, as well as the index terms used to describe those articles. Insights from this analysis informed the development of a comprehensive search strategy incorporating the identified keywords and index terms, as outlined in Multimedia Appendix 1. The final search was conducted by a health librarian (CC-A) in the MEDLINE database via Ovid, covering all records from database inception through May 3, 2024.

#### Expert Opinion Study

Experts in anatomy and related health sciences were recruited through personal invitations extended via the professional networks of the research team. We aimed to engage a total of 6 experts—3 male and 3 female individuals—to ensure balanced representation and to capture a diverse range of perspectives across relevant areas of expertise.

#### Survey

The anonymous survey was conducted on Prolific [13], with a recruitment goal of 300 participants—an approximate sample size considered adequate for robust descriptive analyses in survey research [14]. Eligible individuals from the Prolific participant pool were able to self-select into the study if they met the inclusion criteria. Prior to beginning the 5-minute survey, participants provided electronic informed consent through the platform. Each participant received £0.75 (US $1) as compensation for their time.

### Assessments

#### Review

All identified citations were imported into EndNote (version 21.2; Clarivate Analytics), where duplicates were identified and removed. The unique records were then transferred to Rayyan [15] for study selection. The selection process involved full-text screening of all articles, including appendices, to detect images of PD body charts. Each record was independently assessed by one reviewer (CM or SS), with 10% of excluded articles cross-checked by a second reviewer (SS or CM) according to predefined eligibility criteria. Discrepancies were resolved through discussion between the two reviewers or, if needed, by consultation with a third reviewer (JL-A or CMW). Articles that mentioned the use of PD body charts but did not include images were only retained if the type of body chart used was explicitly referenced and the images could be located through the cited source.

Data were extracted from papers included in the scoping review using the data extraction tool listed in Multimedia Appendix 2. This tool was piloted with 8 articles to ensure its reliability and comprehensiveness. Two reviewers (SS and CM) independently extracted data from the first 10 articles to establish consistency. For the remaining articles, one reviewer (either CM or SS) extracted the data, while a second reviewer cross-verified 10% of the extracted data to maintain quality control. The extracted data included specific details about the populations, concepts, contexts, and study methods relevant to the scoping review’s questions and objectives. Any disagreements between the reviewers were resolved through discussion or, when necessary, by consulting a third reviewer (JL-A or CMW).

#### Expert Opinion Study

Following data extraction, our experts (GC, BAL, SM, LN, GWK, and CJL) independently assessed the sex representation of the PD body charts identified in the scoping review. Images of the body charts were compiled in a Microsoft Excel file for review. Experts were asked to classify each body chart by selecting one of the following predefined categories from a drop-down menu: “Male,” “Female,” “Sex-neutral,” “Male and female (>1 template),” or “Unclear.” As the original articles rarely provided detailed descriptions of the body charts, there were no predefined correct or incorrect answers; rather, the goal was to capture the experts’ subjective assessments informed by their anatomical expertise. In cases where the same body chart appeared multiple times—occasionally in slightly different versions—experts were instructed to assess each version individually. When a body chart included multiple figures with different sex representations, the category “Male and female (>1 template)” was used, where applicable. If none of the predefined categories were suitable, experts had the option to elaborate in a designated comments field.

#### Survey

The survey aimed to examine participants’ perceptions of body charts, focusing specifically on how they classified the chart designed by Lukic et al [10] (as female, male, or sex-neutral), their preferences for selecting a body chart to represent their pain (female, male, or sex-neutral), and the importance they placed on having the option to select from these versions. Additionally, we collected self-reported demographic information, including age, current and past experiences with back pain, country of residence, sex assigned at birth, gender identity, educational attainment, and race. The survey also included additional questions related to the acceptability and usability of the body chart; however, these were not related to the research questions addressed in this paper and were analyzed separately [10].

### Analyses

### Review

We conducted a descriptive analysis of the extracted data, focusing on the geographical location of the studies, whether the sex of the body chart used was reported, and whether single or multiple body charts were used. We also assessed whether the studies reported the sex of the participants and, based on this information, whether they included both male and female participants, or exclusively one sex.

### Expert Opinion Study

Due to the imbalanced distribution of ratings—51.2% of which fell into the “Male” category—both Fleiss κ and Gwet AC1 were calculated to assess inter-rater agreement. Fleiss κ provides a conservative estimate that accounts for prevalence effects, representing a “worst-case” scenario, while the Gwet AC1 offers a more robust measure in the presence of imbalanced data, reflecting a “best-case” scenario [16]. While Fleiss κ is a widely used standard metric, the Gwet AC1 is increasingly recommended for its reliability under uneven category distributions. Agreement levels for both statistics were interpreted using the guidelines established by Landis and Koch [17]. For additional context, raw percentage agreement was also calculated as a straightforward, unadjusted measure of observed consensus among raters. Analyses were performed using the *irr* (version 0.84.1), *irrCAC* (version 1.0), and *DescTools* (version 0.99.59) packages in R (R Foundation for Statistical Computing). Agreement was assessed across 5 categories reflecting experts’ judgments of the body chart’s sex: “Male,” “Female,” “Sex-neutral,” “Male and female (>1 template),” and “Unclear.” The analyses were performed for all raters collectively, as well as separately for female raters, male raters, and for all raters excluding one identified as a potential outlier based on preliminary rating patterns. Additionally, we analyzed majority ratings, defined as instances where at least 4 out of 6 raters assigned the same rating. We also evaluated the extent to which expert ratings aligned with explicit body chart descriptions reported in the literature. Finally, we investigated whether the sex classification assigned by experts corresponded with the sex of the study participants as reported in the articles, considering body charts rated as “sex-neutral” to be a match for participants of any sex.

### Survey

Descriptive statistics were computed for the entire sample, followed by subgroup analyses to compare responses across sex, age groups, back pain experience (current or past), education level, and race. Chi-square tests were conducted to assess whether the distribution of responses differed significantly between subgroups. For the subgroup analyses, dichotomous groups were formed where appropriate: For age, participants were categorized into 2 groups based on the mean age: those aged older than 46 years (above the mean) and those aged 46 years or younger. Educational levels were categorized into “Non-tertiary education” (including “No formal qualification,” “Lower secondary qualification,” “Upper secondary qualification without university access,” and “University entry qualification”) and “Tertiary education” (including “Post-secondary below bachelor’s level,” “Bachelor’s level,” and “Master’s level and above”). Race was grouped into “Largest racial group” (participants identifying as White) and “Other racial groups” (including those identifying as Asian, Black, Latino or Hispanic, Middle Eastern, Indigenous, Aboriginal or First Nations, or “Other,” including multiracial participants). Participants who selected “I prefer not to answer” were excluded from race-based subgroup analyses. Due to the small number of participants with diverse gender identities, subgroup analyses by gender were not feasible. For descriptive purposes, free-text responses indicating “female” or “woman” were categorized as “woman,” and those indicating “male” or “man” as “man.” Responses unrelated to gender identity (eg, referring to sexual orientation) were classified as “other.”

## Results

### Scoping Review

**Figure 1.**
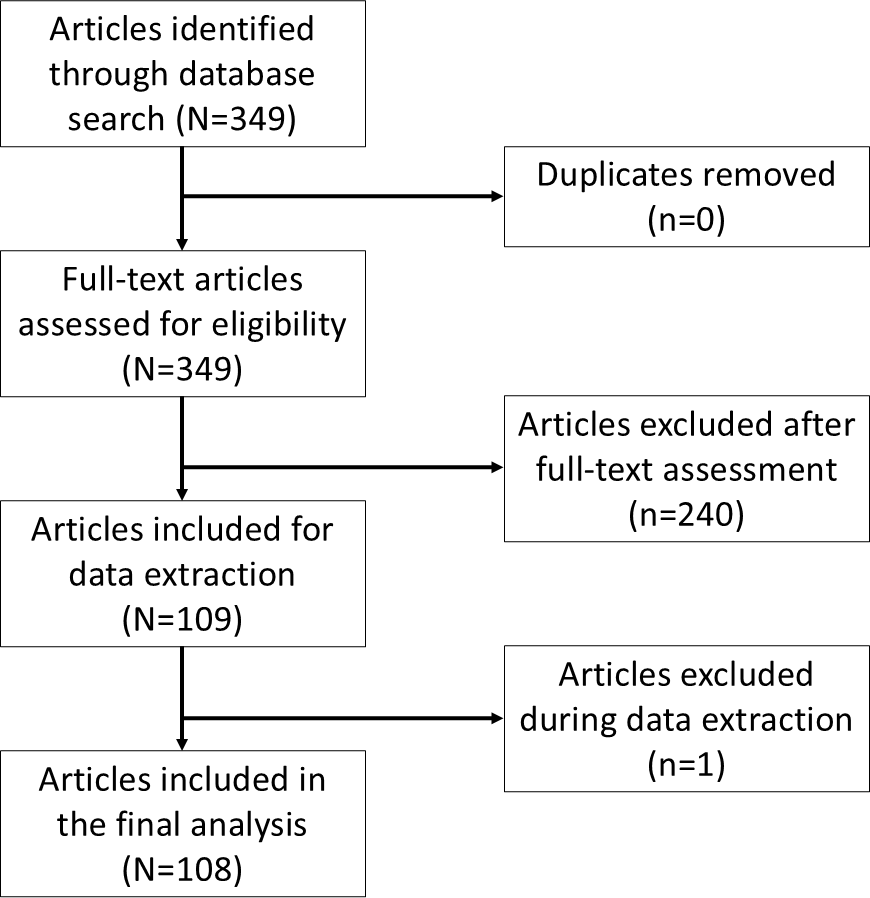
Flowchart of literature search and study selection

Our search yielded 349 articles, of which 109 were selected for further analysis after full-text screening. During data extraction, one article was excluded because it used actual participant photos instead of an illustrated body chart for PD, resulting in a final sample of 108 articles (see Figure 1 and full citations in Multimedia Appendix 3). The articles referred to studies predominantly conducted in Europe (n=60, 55.6%) and North America (n=31, 28.7%) and were published between 1988 and 2022. Notably, none of the articles explicitly stated an intention to use a sex-neutral body chart, and only 5 (4.6%) articles reported the sex of the body chart used. Among these, 4 articles [8,18-20] used 2 different body charts described as male and female, while 1 article [21] used a single body chart described as male. Out of the 108 articles, 6 (5.6%) used 2 different body charts [3,8,18-20,22], while the remaining 102 (94.4%) used a single body chart. One of the studies using two charts included only female participants [22]. Among studies using a single body chart, 70.6% (72/102) included both male and female participants [4,21,23-92], 8.8% (9/102) included only female participants [93-101], and 2.0% (2/102) included only male participants [102,103]. In 18.6% (19/102) of studies, the sex or gender of participants was not clearly reported; these studies used general terms such as “patients,” “subjects,” or “employees,” without further specification [104-122].

### Expert Opinion Study

**Figure 2.**
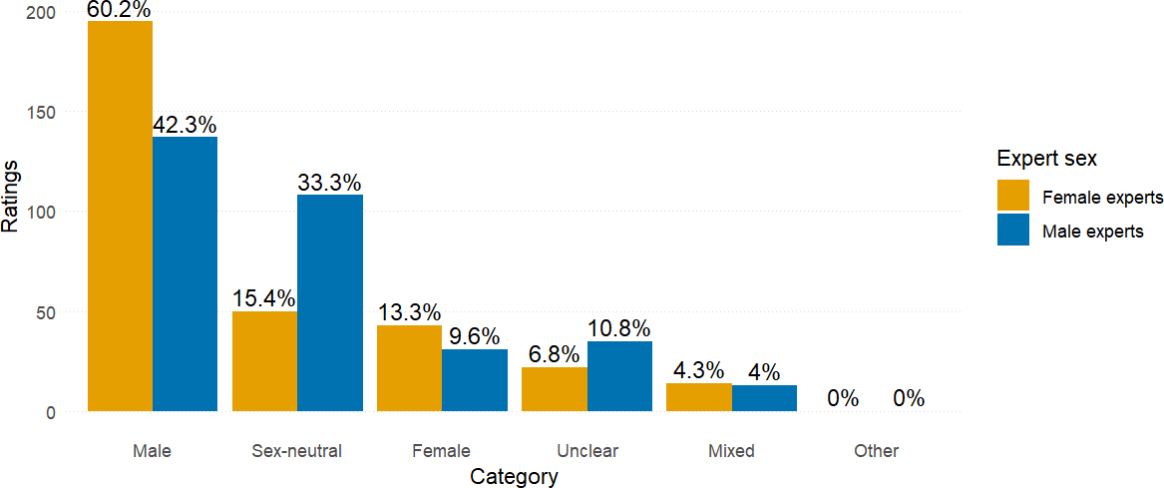
Body chart ratings by category and expert sex. The categories represent experts’ ratings of the perceived sex of the body charts. “Mixed” refers to body charts that included multiple figures perceived as both male and female. Each group of experts—female and male—contributed 324 ratings, for a total of 648 ratings.

The expert panel consisted of 6 raters—3 female and 3 male raters—including 4 physicians (an orthopedic spine surgeon, a radiologist, a chiropractor, and an anatomy lecturer), an anatomical dissection specialist, and a neuroscientist. All experts self-identified as White. Each expert independently assessed the sex representation of body charts from 108 articles, resulting in a total of 648 individual ratings. The distribution of ratings was as follows: 332 (51.2%) ratings were classified as “male,” 158 (24.4%) as “sex-neutral,” 74 (11.4%) as “female,” 27 (4.2%) as “male and female” (for charts depicting two different bodies), 57 (8.8%) as “unclear,” and 0 (0%) as “other.” Female experts classified 1.42 times more body charts as “male” (195 ratings, 60.2% of their total) compared to male experts (137 ratings, 42.3% of their total). Conversely, male experts assigned more than twice as many body charts as “sex-neutral” (108 ratings, 33.3% of their total) than the female experts (50 ratings, 15.4% of their total). See Figure 2 for a visual representation of these findings.

Table 1 presents the inter-rater agreement metrics for the overall sample and subgroups. Across all raters, the Fleiss κ indicated fair agreement, while the Gwet AC1 suggested moderate agreement. The simple percentage agreement was 10.2% (see Multimedia Appendix 4 for images that were rated identically). Subgroup analyses revealed the following patterns: for the 3 female raters, there was moderate agreement for both Fleiss κ and Gwet AC1, and the simple percentage agreement was 52.8%. The 3 male raters revealed slight agreement for Fleiss κ and fair agreement for Gwet AC1, with one suspected outlier; the simple percentage agreement was 13.9%. After excluding the suspected outlier, agreement among the remaining 5 raters increased to moderate agreement (Fleiss κ) and substantial (Gwet AC1), with a percentage agreement of 49.1%. All interpretations should be considered alongside the confidence intervals in Table 1, and percentage agreement values should be interpreted with caution due to the limited nuance.

**Table 1.** Inter-rater agreement metrics.

Group and metric	Metric value	95% CI	Interpretation
All raters (n=6)			
Fleiss κ	0.306	0.276‐0.336	Fair agreement
Gwet AC1	0.456	0.401‐0.511	Moderate agreement
Percentage agreement	10.2	N/A^a^	—^b^
Female raters (n=3)			
Fleiss κ	0.404	0.337‐0.47	Moderate agreement
Gwet AC1	0.587	0.496‐0.679	Moderate agreement
Percentage agreement	52.8	N/A	—
Male raters (n=3)			
Fleiss κ	0.098	0.031‐0.165	Slight agreement
Gwet AC1	0.251	0.188‐0.314	Fair agreement
Percentage agreement	13.9	N/A	—
Without male outlier (n=5)			
Fleiss κ	0.488	0.452‐0.524	Moderate agreement
Gwet AC1	0.647	0.571‐0.724	Substantial agreement
Percentage agreement	49.1	N/A	—

a N/A: not applicable.

b —: not available.

We also examined majority ratings, defined as at least 4 out of 6 raters assigning the same classification. Body charts were categorized as “male” in more than half of the articles (59/108, 54.6%), “female” in 10 (9.3%) articles, “sex-neutral” in 8 (7.4%) articles, “male and female” in 4 (3.7%) articles, and “unclear” in 1 (0.9%) article. In 26 articles (24.1%), ratings were too inconsistent to achieve a majority.

Of the 108 articles, only 5 explicitly described the body charts as male, female, or sex-neutral. Four of these articles included 2 body charts each, described as “male” and “female.” In three of these articles, all raters classified the sex of the charts in line with the article descriptions. In the fourth, however, rater assessments diverged: four classified both charts as “female,” and two as “sex-neutral.” The fifth article included a single body chart described as male, which 5 experts identified as “male” and one as “unclear.”

Finally, we assessed alignment between the expert-assigned sex of the body charts and the sex of study participants as reported in the articles. Of the 108 articles, 40 (37.0%) provided unclear descriptions of participants’ sex or lacked a majority rating from the experts. In the remaining 68 articles, 22.1% (15/68) showed a match between the chart and participant sex. The majority, 67.7% (46/68), were male-biased (ie, male body charts used in studies not exclusively involving male participants), while 10.3% (7/68) were female-biased.

### Survey

A total of 300 individuals participated in the survey; however, two were excluded due to technical issues, resulting in a final sample of 298 participants. Descriptive statistics for the sample are presented in Table 2, and subgroup comparisons, including chi-square test results, are shown in Table 3. The majority of respondents (204/298, 68.5%) perceived Lukic et al’s [10] body chart as sex-neutral. In contrast, 20.1% (60/298) identified it as representing a male body, while 11.4% (34/298) perceived it as female. Chi-square tests revealed no significant differences in response distributions based on sex, age group, current or past back pain experience, or educational background. However, a significant association was observed for race: participants who identified as White were more likely to perceive the body chart as sex-neutral compared to those who identified as Asian; Black; Latino or Hispanic; Middle Eastern; Indigenous, Aboriginal, or First Nations; or “Other” (Table 3).

**Table 2.** Survey sample characteristics (n=298).

Characteristics	Value
Age (y)	
Mean (SD)	46.16 (15.35)
Min	18
Max	79
Current back pain, n (%)	
Yes	154 (51.7)
Back pain in past, n (%)	
Yes	239 (80.2)
Participants’ sex, n (%)	
Female	154 (51.7)
Male	144 (48.3)
Participants’ gender, n (%)	
Woman	148 (49.7)
Man	133 (44.6)
Other	2 (0.7)
Missing	15 (5)
Education, n (%)	
Nontertiary	134 (45)
No formal qualification	2
Lower secondary qualification	21
Upper secondary qualification without university access	80
University entry qualification	31
Tertiary	164 (55)
Post-secondary below bachelor’s level	10
Bachelor’s level	96
Master’s level and higher	58
Race, n (%)	
Asian	24 (8.1)
Black	12 (4)
Indigenous, Aboriginal, or First Nations	0 (0)
Latino or Hispanic	1 (0.3)
Middle Eastern	6 (2)
Other	6 (2)
White	248 (83.2)
I prefer not to answer	5 (1.7)

**Table 3.** Responses to the question: “Does this body chart represent a female, male, or sex-neutral body?” by participants’ sex, age, back pain experience, education, and race.

Variable	Body chart, n (%)			Chi-square (*df*)	*P* value
	Sex-neutral	Male	Female		
Participants’ sex				0.3 (2)	.92
Female (n=154)	104 (67.5)	31 (20.1)	19 (12.3)		
Male (n=144)	100 (69.4)	29 (20.1)	15 (10.4)		
Age groups (y)				4.3 (2)	.13
≤46 (n=139)	87 (62.6)	34 (24.5)	18 (13)		
>46 (n=159)	117 (73.6)	26 (16.4)	16 (10.1)		
Current back pain				0.9 (2)	.66
No (n=144)	97 (67.4)	32 (22.2)	15 (10.4)		
Yes (n=154)	107 (69.5)	28 (18.2)	19 (12.3)		
Past back pain				1.1 (2)	.57
No (n=59)	38 (64.4)	12 (20.3)	9 (15.3)		
Yes (n=239)	166 (69.5)	48 (20.1)	25 (10.5)		
Educational attainment				1.0 (2)	.61
Nontertiary (n=134)	93 (69.4)	24 (17.9)	17 (12.7)		
Tertiary (n=164)	111 (67.7)	36 (22)	17 (10.4)		
Race				15.9 (2)	.001
Other racial groups^a^ (n=40)	17 (42.5)	13 (32.5)	10 (25)		
Largest racial group^b^ (n=248)	181 (73)	44 (17.7)	23 (9.3)		

a “Other racial groups” includes participants who identified as Asian, Black, Middle Eastern, Latino or Hispanic, Indigenous, Aboriginal, First Nations, or “Other” (including multiracial).

b “Largest racial group” includes participants who identified as White.

Most participants (189/298, 63.4%) indicated that it was not important to them to be able to choose between female, male, or sex-neutral versions of the body chart. This included 53.3% (82/154) of female participants and 74.3% (107/144) of male participants. When given the option, over half of the female participants (85/154; 55.2%) said they would choose a female body chart, while 46.1% (71/154) preferred a sex-neutral body chart, and 2 (1.3%) selected a male body chart. In contrast, nearly two-thirds of male participants (88/144, 61.1%) preferred a sex-neutral body chart, 36.1% (52/144) chose a male body chart, 2.8% (4/144) opted for a female body chart. Two participants did not identify with the sex assigned to them at birth; both were assigned male and identified as women. One rated the body chart as male, preferred a female body chart, and expressed that having the option to choose is important. The other rated the chart as sex-neutral, preferred a sex-neutral chart, and reported that the option to choose is not important. Due to the small number of participants with diverse gender identities, no conclusions can be drawn regarding their perceptions and preferences.

## Discussion

### Principal Results

Our review revealed that the vast majority of articles in the back pain literature do not explicitly report the sex of the body chart used, nor do they indicate an intention of sex neutrality. Very few articles included both male and female versions of body charts. In our expert opinion study, over half of the body charts were classified as “male” and did not accurately reflect the sex of the study participants. Specifically, two-thirds were identified as male-biased—meaning male body charts were used in studies that did not exclusively involve male participants. Agreement among expert raters ranged from fair to moderate, with stronger agreement observed among female raters than male raters. This suggests that assessing the sex of body charts is inherently challenging. While many charts may have been designed to be sex-neutral, they are not consistently perceived as such. In contrast, Lukic et al’s [10] body chart—explicitly designed as sex-neutral by an interdisciplinary team—was perceived as sex-neutral by two-thirds of male and female survey participants, regardless of age, back pain experience (current or past), or education level. However, a significant difference emerged by race: participants who identified as White were more likely to perceive the chart as sex-neutral compared to those who did not identify as White. In terms of preference, female participants showed a slight inclination toward female body charts over sex-neutral ones, while male participants clearly preferred sex-neutral charts over male ones. Notably, both male and female participants generally considered the ability to choose among body chart versions to be of little importance. These findings suggest that a well-designed sex-neutral body chart is broadly accepted by both male and female users and holds the potential for promoting greater sex and gender inclusivity in PDs.

### Comparison With Prior Work

Despite the introduction of sex-specific PD body charts in the late 1980 s [3], our findings show that most subsequent studies have continued to use a single body chart—typically perceived as male—to represent both male and female participants. This supports previous observations noting that most PD body charts used in the literature are male [1,7]. To more accurately reflect the diversity of study participants, future studies could either provide different versions of body charts that align with the sex or gender of participants [8,19,20] or offer a sex-neutral version that is broadly perceived as truly neutral [10].

The variability in expert ratings underscores the subjectivity involved in perceiving sex representations. What one expert identifies as male, female, or sex-neutral may differ from another’s interpretation, even when both have similar anatomical training and expertise. This echoes earlier findings showing variability in how study participants perceive sex in body charts [7]. Such discrepancies may stem from individual experiences and implicit biases, as well as from the design of body charts that omit explicit sex-specific cues. While the absence of these cues is likely intended to enhance neutrality, it may also make interpretation difficult, leading to the fair to moderate agreement levels observed in our study. This suggests that many of the body charts found in the literature were likely intended to be sex-neutral but were not consistently interpreted as such—instead, they are most often interpreted as “male.”

A male bias has been well-documented in human sex perception and anatomical illustrations. Research indicates that when faced with ambiguity or uncertainty, humans are more likely to perceive a figure as male [123,124]. This perceptual bias may have evolutionary roots: misidentifying a potentially dangerous individual (typically male) as female could have higher survival costs, aligning with instinctual self-preservation mechanisms [123]. Therefore, “male” often becomes the default in sex perception, while “female” is only assumed when explicit female sex cues are present [124]. This phenomenon aligns with our findings and may help explain why body charts with ambiguous or unclear sex cues were predominantly perceived as male. Additionally, the design of the body charts themselves—even when designed to be sex-neutral—may reflect a bias toward male figures. Such bias is not unique to PDs but mirrors a broader trend in anatomical illustrations, where male figures are overrepresented, reinforcing the male body as the normative standard [125-129].

Given this observed male bias and the imbalance in sex representation, it is essential to consider the needs and preferences of individuals currently underrepresented in PD tools. Our survey results suggest that a truly sex-neutral body chart is well-accepted by both male and female individuals. While female individuals showed a slight preference for a female-specific body chart, the option to choose between different body chart versions was generally not considered crucial. This nuance builds on the findings of Egsgaard et al [1], who reported a preference among female participants for female body charts. It is important to note, however, that participants in Egsgaard et al’s study [1] were only presented with male and female options—without the inclusion of a sex-neutral alternative. Interestingly, while female participants in that study expressed a clear preference for female body charts over male ones, more than half indicated that the ability to choose was not particularly important. This aligns with our results, suggesting that although preferences exist, they may not be a crucial factor in pain reporting. The methodological difference in body chart options presented likely accounts for the partial discrepancy in findings between our study and the study by Egsgaard et al [1]. Together, these insights highlight the potential value of including truly sex-neutral body charts in PD tools, as they may offer a more inclusive and representative option for diverse patient populations while still accommodating those with a preference for sex-specific representations.

### Limitations

Our scoping review aimed for comprehensiveness but was limited by language restrictions (English and German) and the exclusive use of the MEDLINE database. These constraints may have introduced selection bias, potentially excluding relevant literature and culturally specific body charts. Future research should expand language criteria and database inclusion to ensure a more comprehensive review. Another potential limitation is that some articles may have displayed only a single body chart, despite using multiple charts in their study. This could have led to misinterpretations and inaccurate conclusions in our review.

Regarding the expert assessment, an important limitation was the lack of cultural diversity among the experts, as all experts self-identified as White. Future studies should aim to include a more diverse panel to assess how cultural backgrounds might influence perceptions of the body charts.

Similarly, of the 298 survey participants, only 40 did not identify as White, which limits the generalizability of our findings to racially diverse populations. Despite this, the survey sample was broad in terms of age and educational background and approximately balanced in terms of sex. Future research should explore how the body chart designed by Lukic et al [10] is perceived by individuals from a broader range of racial and cultural backgrounds to better understand its cross-cultural applicability and inclusivity.

### Clinical Implications

The observed acceptance of our sex-neutral body chart across sexes highlights the potential of sex-neutral PD tools to offer a more universally applicable approach to pain assessment. These tools may be particularly valuable in clinical settings where traditional sex-specific charts are impractical or where sex information is unavailable or deemed nonessential—for example, in emergency care settings, large-scale epidemiological studies, or in the care of nonbinary and transgender individuals. This approach aligns with a growing shift toward inclusivity in medical tools, such as the development of non–sex-specific growth charts for transgender youth [125]. Moreover, sex-neutral PD tools could be particularly beneficial in digital health applications, where standardized, sex-neutral inputs would facilitate automated analysis and improve consistency across diverse populations.

### Conclusions

Our scoping review and subsequent studies reveal important gaps and biases in the reporting and representation of sex in PD body charts used in back pain research. The majority of charts are perceived as male, often misrepresenting the study population they are intended to depict. Expert assessments showed fair to moderate agreement, suggesting the complexity of sex perception and indicating that many charts—despite possibly being intended as sex-neutral—are not consistently perceived as such. Our study offers a promising solution: a newly designed sex-neutral body chart, developed with expert input, was widely perceived as sex-neutral across diverse participant groups. However, variations in perception across racial groups highlight the importance of incorporating cultural considerations into design. These findings underscore the potential of a truly sex-neutral body chart to support greater sex and gender balance in pain assessment tools. Future research should prioritize the development and evaluation of culturally sensitive body chart designs to further promote inclusivity and equity in both back pain research and clinical practice.

## Supplementary material

10.2196/76175 Multimedia Appendix 1 Search strategy.

10.2196/76175 Multimedia Appendix 2 Data extraction tool.

10.2196/76175 Multimedia Appendix 3 References to included studies.

10.2196/76175 Multimedia Appendix 4 Body charts with 100% agreement among all 6 experts.

## Abbreviations

PD: pain drawing
SAGER: Sex and Gender Equity in Research
