# Supplementary material for: Sex Representation and User Preferences in Pain Drawing Body Charts in Back Pain Research: Multimethod Study
Source: JMIR Hum Factors. 2026 Feb 9;13:e76175. doi: 10.2196/76175 (PMC12885194; doi:10.2196/76175)
Supplement: Multimedia Appendix 1 [file humanfactors-v13-e76175-s001.docx]

**Multimedia Appendix 1. Search Strategy**

The strategy for the MEDLINE (Ovid) search conducted on 3 May 2024 was as follows:

| 1 | ("backpain" or "neck pain" or backache or lumbago or "neck ache" or "spin* pain" or "sciatica" or "painfuly back" or ("back" adj2 "pain") or ("lumbar" adj2 "pain") or "back disorder*").mp. | 101877 |
| --- | --- | --- |
| 2 | exp Back Pain/ or exp sciatic neuropathy/ or exp neck pain/ | 59735 |
| 3 | 1 or 2 | 104431 |
| 4 | ((("Pain" or "symptom*" or "painful*" or "body") adj ("drawing*" or "representation*" or "Painting*" or "illustration*" or "Illustrative*" or "diagram*" or "map*" or "area*" or "chart*" or "outline*")) or "body Surface" or "body distribution" or "manikin*" or "mannequin*").mp. | 50285 |
| 5 | exp pain/ and (exp Models, Anatomic/ or Medical Illustration/) | 275 |
| 6 | 4 or 5 | 50511 |
| 7 | ("Pain Perception*" or "pain measurement" or "pain distribution" or "pain assessment" or "pain location" or "painful location" or "pain area" or "painful area" or "pain sites" or "painful sites" or "pain area" or "self-reported pain" or ("pain" adj "diagnos*")).mp. or exp Musculoskeletal Pain/di or exp Pain Measurement or exp Pain Perception/ | 114397 |
| 8 | 3 and 6 and 7 | 356 |
| 9 | 8 and (english or german).lg. | 349 |
