# Supplementary material for: Sex Representation and User Preferences in Pain Drawing Body Charts in Back Pain Research: Multimethod Study
Source: JMIR Hum Factors. 2026 Feb 9;13:e76175. doi: 10.2196/76175 (PMC12885194; doi:10.2196/76175)
Supplement: Multimedia Appendix 3 [file humanfactors-v13-e76175-s003.docx]

**Multimedia Appendix 3. References to Included Studies**

**Included Studies (N = 108)**

The literature search yielded 349 articles, all of which underwent full-text screening. After full-text screening and data extraction, 108 articles were included in the final analysis [1-108].

**References**

1. Abbott JH, Foster M, Hamilton L, Ravenwood M, Tan N. Validity of pain drawings for predicting psychological status outcome in patients with recurrent or chronic low back pain. The Journal of manual & manipulative therapy. 2015;23(1):12-19. doi: 10.1179/2042618613Y.0000000046.

2. Abichandani D, Barbero M, Cescon C, Gallace A, Punt D, Sanchis-Sanchez E, Falla D. Can People with Chronic Neck Pain Recognize Their Own Digital Pain Drawing? Pain physician. 2020;23(2):E231-E240. PMID: 32214308.

3. Albert HB, Hansen JK, Sogaard H, Kent P. Where do patients with MRI-confirmed single-level radiculopathy experience pain, and what is the clinical interpretability of these pain patterns? A cross-sectional diagnostic accuracy study. Chiropractic & manual therapies. 2019;27:50. doi: 10.1186/s12998-019-0273-8.

4. Alev L, Fujikoshi S, Yoshikawa A, Enomoto H, Ishida M, Tsuji T, et al. Duloxetine 60 mg for chronic low back pain: post hoc responder analysis of double-blind, placebo-controlled trials. Journal of pain research. 2017;10:1723-1731. doi: 10.2147/JPR.S138297.

5. Alter BJ, Anderson NP, Gillman AG, Yin Q, Jeong J-H, Wasan AD. Hierarchical clustering by patient-reported pain distribution alone identifies distinct chronic pain subgroups differing by pain intensity, quality, and clinical outcomes. PloS one. 2021;16(8):e0254862. doi: 10.1371/journal.pone.0254862.

6. Andersen T, Christensen FB, Hansen ES, Bunger C. Pain 5 years after instrumented and non-instrumented posterolateral lumbar spinal fusion. European spine journal : official publication of the European Spine Society, the European Spinal Deformity Society, and the European Section of the Cervical Spine Research Society. 2003;12(4):393-399. doi: 10.1007/s00586-003-0547-6.

7. Andersen T, Christensen FB, Hoy KW, Helmig P, Niedermann B, Hansen ES, Bunger C. The predictive value of pain drawings in lumbar spinal fusion surgery. The spine journal : official journal of the North American Spine Society. 2010;10(5):372-379. doi: 10.1016/j.spinee.2010.02.002.

8. Apeldoorn AT, Bosselaar H, Ostelo RW, Blom-Luberti T, van der Ploeg T, Fritz JM, et al. Identification of patients with chronic low back pain who might benefit from additional psychological assessment. The Clinical journal of pain. 2012;28(1):23-31. doi: 10.1097/AJP.0b013e31822019d0.

9. Barbero M, Moresi F, Leoni D, Gatti R, Egloff M, Falla D. Test-retest reliability of pain extent and pain location using a novel method for pain drawing analysis. European journal of pain (London, England). 2015;19(8):1129-1138. doi: 10.1002/ejp.636.

10. Barbero M, Piff M, Evans D, Falla D. Do metrics derived from self-reported and clinician-reported pain drawings agree for individuals with chronic low back pain? Musculoskeletal science & practice. 2023;68:102871. doi: 10.1016/j.msksp.2023.102871.

11. Bertilson BC, Brosjo E, Billing H, Strender L-E. Assessment of nerve involvement in the lumbar spine: agreement between magnetic resonance imaging, physical examination and pain drawing findings. BMC musculoskeletal disorders. 2010;11:202. doi: 10.1186/1471-2474-11-202.

12. Bishop MD, Horn ME, George SZ, Robinson ME. Self-reported pain and disability outcomes from an endogenous model of muscular back pain. BMC musculoskeletal disorders. 2011;12:35. doi: 10.1186/1471-2474-12-35.

13. Bishop MD, Horn ME, Lott DJ, Arpan I, George SZ. Magnitude of spinal muscle damage is not statistically associated with exercise-induced low back pain intensity. The spine journal : official journal of the North American Spine Society. 2011;11(12):1135-1142. doi: 10.1016/j.spinee.2011.11.005.

14. Boudreau SA, Badsberg S, Christensen SW, Egsgaard LL. Digital pain drawings: assessing touch-screen technology and 3D body schemas. The Clinical journal of pain. 2016;32(2):139-145. doi: 10.1097/AJP.0000000000000230.

15. Burkey AR, Kanetsky PA. Development of a novel location-based assessment of sensory symptoms in cancer patients: preliminary reliability and validity assessment. Journal of pain and symptom management. 2009;37(5):848-862. doi: 10.1016/j.jpainsymman.2008.05.013.

16. Falla D, Peolsson A, Peterson G, Ludvigsson ML, Soldini E, Schneebeli A, Barbero M. Perceived pain extent is associated with disability, depression and self-efficacy in individuals with whiplash-associated disorders. European journal of pain (London, England). 2016;20(9):1490-1501. doi: 10.1002/ejp.873.

17. Friedrich M, Gittler G, Pieler-Bruha E. Misleading history of pain location in 51 patients with osteoporotic vertebral fractures. European spine journal : official publication of the European Spine Society, the European Spinal Deformity Society, and the European Section of the Cervical Spine Research Society. 2006;15(12):1797-1800. doi: 10.1007/s00586-006-0065-4.

18. Hagg O, Fritzell P, Hedlund R, Moller H, Ekselius L, Nordwall A. Pain-drawing does not predict the outcome of fusion surgery for chronic low-back pain: a report from the Swedish Lumbar Spine Study. European spine journal : official publication of the European Spine Society, the European Spinal Deformity Society, and the European Section of the Cervical Spine Research Society. 2003;12(1):2-11. doi: 10.1007/s00586-002-0427-5.

19. Chan CW, Goldman S, Ilstrup DM, Kunselman AR, O'Neill PI. The pain drawing and Waddell's nonorganic physical signs in chronic low-back pain. Spine. 1993;18(13):1717-1722. doi: 10.1097/00007632-199310000-00001.

20. Clark EM, Hutchinson AP, McCloskey EV, Stone MD, Martin JC, Bhalla AK, Tobias JH. Lateral back pain identifies prevalent vertebral fractures in post-menopausal women: cross-sectional analysis of a primary care-based cohort. Rheumatology (Oxford, England). 2010;49(3):505-512. doi: 10.1093/rheumatology/kep414.

21. Cleland JA, Childs JD, Palmer JA, Eberhart S. Slump stretching in the management of non-radicular low back pain: a pilot clinical trial. Manual therapy. 2006;11(4):279-286. doi: 10.1016/j.math.2005.07.002.

22. Correa LA, Bittencourt JV, Ferreira AdS, Reis FJJD, de Almeida RS, Nogueira LAC. The reliability and concurrent validity of painMAP software for automated quantification of pain drawings on body charts of patients with low back pain. Pain practice : the official journal of World Institute of Pain. 2020;20(5):462-470. doi: 10.1111/papr.12872.

23. Cruder C, Falla D, Mangili F, Azzimonti L, Araújo LS, Williamon A, Barbero M. Profiling the location and extent of musicians’ pain using digital pain drawings. Pain Practice. 2018 2018/01/01;18(1):53-66. PMID: 28466572. doi: 10.1111/papr.12581.

24. De Oliveira Paes Leme M, Yuan SLK, Oliveira Magalhaes M, Ferreira de Meneses SR, Marques AP. Pain and quality of life in knee osteoarthritis, chronic low back pain and fibromyalgia: a comparative cross-sectional study. Reumatismo. 2019;71(2):68-74. doi: 10.4081/reumatismo.2019.1104.

25. Dean LE, Arnold L, Crofford L, Bennett R, Goldenberg D, Fitzcharles M-A, et al. Impact of moving from a widespread to multisite pain definition on other fibromyalgia symptoms. Arthritis care & research. 2017;69(12):1878-1886. doi: 10.1002/acr.23214.

26. Dyrehag LE, Widerstrom-Noga EG, Carlsson SG, Kaberger K, Hedner N, Mannheimer C, Andersson SA. Relations between self-rated musculoskeletal symptoms and signs and psychological distress in chronic neck and shoulder pain. Scandinavian journal of rehabilitation medicine. 1998;30(4):235-242. PMID: 9825388.

27. Ekman P, Moller H, Hedlund R. Predictive factors for the outcome of fusion in adult isthmic spondylolisthesis. Spine. 2009;34(11):1204-1210. doi: 10.1097/BRS.0b013e3181a19e66.

28. Fukui S, Ohseto K, Shiotani M. Patterns of pain induced by distending the thoracic zygapophyseal joints. Regional anesthesia. 1997;22(4):332-336. doi: 10.1016/s1098-7339(97)80007-7.

29. Galve Villa M, Palsson TS, Boudreau SA. Spatiotemporal patterns of pain distribution and recall accuracy: a dose-response study. Scandinavian journal of pain. 2022;22(1):154-166. doi: 10.1515/sjpain-2021-0032.

30. Georgopoulos V, Akin-Akinyosoye K, Smith S, McWilliams DF, Hendrick P, Walsh DA. An observational study of centrally facilitated pain in individuals with chronic low back pain. Pain reports. 2022;7(3):e1003. doi: 10.1097/PR9.0000000000001003.

31. Gerhardt A, Eich W, Janke S, Leisner S, Treede R-D, Tesarz J. Chronic widespread back pain is distinct from chronic local back pain: evidence from quantitative sensory testing, pain drawings, and psychometrics. The Clinical journal of pain. 2016;32(7):568-579. doi: 10.1097/AJP.0000000000000300.

32. Gerhardt A, Eich W, Treede R-D, Tesarz J. Conditioned pain modulation in patients with nonspecific chronic back pain with chronic local pain, chronic widespread pain, and fibromyalgia. Pain. 2017;158(3):430-439. doi: 10.1097/j.pain.0000000000000777.

33. Gerhardt A, Hartmann M, Blumenstiel K, Tesarz J, Eich W. The prevalence rate and the role of the spatial extent of pain in nonspecific chronic back pain--a population-based study in the south-west of Germany. Pain medicine (Malden, Mass). 2014;15(7):1200-1210. doi: 10.1111/pme.12286.

34. Goldstein P, Ashar Y, Tesarz J, Kazgan M, Cetin B, Wager TD. Emerging clinical technology: application of machine learning to chronic pain assessments based on emotional body maps. Neurotherapeutics : the journal of the American Society for Experimental NeuroTherapeutics. 2020;17(3):774-783. doi: 10.1007/s13311-020-00886-7.

35. Grunnesjo M, Bogefeldt J, Blomberg S, Delaney H, Svardsudd K. The course of pain drawings during a 10-week treatment period in patients with acute and sub-acute low back pain. BMC musculoskeletal disorders. 2006;7:65. doi: 10.1186/1471-2474-7-65.

36. Hartvigsen J, Davidsen M, Hestbaek L, Sogaard K, Roos EM. Patterns of musculoskeletal pain in the population: a latent class analysis using a nationally representative interviewer-based survey of 4817 Danes. European journal of pain (London, England). 2013;17(3):452-460. doi: 10.1002/j.1532-2149.2012.00225.x.

37. Hayashi K, Arai Y-CP, Morimoto A, Aono S, Yoshimoto T, Nishihara M, et al. Associations between pain drawing and psychological characteristics of different body region pains. Pain practice : the official journal of World Institute of Pain. 2015;15(4):300-307. doi: 10.1111/papr.12173.

38. Jefferson JR, McGrath PJ. Back pain and peripheral joint pain in an industrial setting. Archives of physical medicine and rehabilitation. 1996;77(4):385-390. doi: 10.1016/s0003-9993(96)90089-1.

39. Kapitza C, Ludtke K, Tampin B, Ballenberger N. Application and utility of a clinical framework for spinally referred neck-arm pain: A cross-sectional and longitudinal study protocol. PloS one. 2020;15(12):e0244137. doi: 10.1371/journal.pone.0244137.

40. Kennedy DJ, Mattie R, Nguyen Q, Hamilton S, Conrad B. Glenohumeral joint pain referral patterns: a descriptive study. Pain medicine (Malden, Mass). 2015;16(8):1603-1609. doi: 10.1111/pme.12797.

41. Hildebrandt J, Franz CE, Choroba-Mehnen B, Temme M. The use of pain drawings in screening for psychological involvement in complaints of low-back pain. Spine. 1988;13(6):681-685.

42. Hoshino H, Sasaki N, Ide K, Yamato Y, Watanabe Y, Matsuyama Y. Effect of central sensitization inventory on the number of painful sites and pain severity in a Japanese regional population cohort. Journal of orthopaedic science : official journal of the Japanese Orthopaedic Association. 2022;27(4):929-934. doi: 10.1016/j.jos.2021.05.003.

43. Hsieh P-H, Chang Y, Chen DW, Lee MS, Shih H-N, Ueng SWN. Pain distribution and response to total hip arthroplasty: a prospective observational study in 113 patients with end-stage hip disease. Journal of orthopaedic science : official journal of the Japanese Orthopaedic Association. 2012;17(3):213-218. doi: 10.1007/s00776-012-0204-1.

44. Huang P, Sengupta DK. How fast pain, numbness, and paresthesia resolves after lumbar nerve root decompression: a retrospective study of patient's self-reported computerized pain drawing. Spine. 2014;39(8):E529-536. doi: 10.1097/BRS.0000000000000240.

45. Hullemann P, Keller T, Kabelitz M, Freynhagen R, Tolle T, Baron R. Pain drawings improve subgrouping of low back pain patients. Pain practice : the official journal of World Institute of Pain. 2017;17(3):293-304. doi: 10.1111/papr.12470.

46. Killinger KA, Boura JA, Peters KM. Pain in interstitial cystitis/bladder pain syndrome: do characteristics differ in ulcerative and non-ulcerative subtypes? International urogynecology journal. 2013;24(8):1295-1301. doi: 10.1007/s00192-012-2003-9.

47. Kloimstein H, Likar R, Kern M, Neuhold J, Cada M, Loinig N, et al. Peripheral nerve field stimulation (PNFS) in chronic low back pain: a prospective multicenter study. Neuromodulation : journal of the International Neuromodulation Society. 2014;17(2):180-187. doi: 10.1111/ner.12139.

48. Knox PJ, Simon CB, Pohlig RT, Pugliese JM, Coyle PC, Sions JM, Hicks GE. Movement-evoked pain versus widespread pain: a longitudinal comparison in older adults with chronic low back pain from the Delaware spine studies. The journal of pain. 2023;24(6):980-990. doi: 10.1016/j.jpain.2023.01.012.

49. Lorenc T, Golebiowski M, Syganiec D, Glinkowski WM. Associations between patient report of pain and intervertebral foramina changes visible on axial-loaded lumbar magnetic resonance imaging. Diagnostics (Basel, Switzerland). 2022;12(3). doi: 10.3390/diagnostics12030563.

50. Lam K, Peolsson A, Soldini E, Lofgren H, Wibault J, Dedering A, et al. Larger pain extent is associated with greater pain intensity and disability but not with general health status or psychosocial features in patients with cervical radiculopathy. Medicine. 2021;100(8):e23718. doi: 10.1097/MD.0000000000023718.

51. Larsen LH, Hirata RP, Graven-Nielsen T. Reorganized trunk muscle activity during multidirectional floor perturbations after experimental low back pain: a comparison of bilateral versus unilateral pain. The journal of pain. 2016;17(2):223-235. doi: 10.1016/j.jpain.2015.10.012.

52. Larsen LH, Hirata RP, Graven-Nielsen T. Pain-evoked trunk muscle activity changes during fatigue and DOMS. European journal of pain (London, England). 2017;21(5):907-917. doi: 10.1002/ejp.993.

53. Lee H, Nicholoson LL, Adams RD, Bae S-S. Body chart pain location and side-specific physical impairment in subclinical neck pain. Journal of manipulative and physiological therapeutics. 2005;28(7):479-486. doi: 10.1016/j.jmpt.2005.07.004.

54. MacDowall A, Robinson Y, Skeppholm M, Olerud C. Anxiety and depression affect pain drawings in cervical degenerative disc disease. Upsala journal of medical sciences. 2017;122(2):99-107. doi: 10.1080/03009734.2017.1319441.

55. Mann NH, 3rd, Brown MD, Enger I. Expert performance in low-back disorder recognition using patient pain drawings. Journal of spinal disorders. 1992;5(3):254-259. doi: 10.1097/00002517-199209000-00002.

56. Masferrer R, Prendergast V, Hagell P. Colored pain drawings: preliminary observations in a neurosurgical practice. European journal of pain (London, England). 2003;7(3):213-217. doi: 10.1016/S1090-3801(02)00113-1.

57. McCormick ZL, Sperry BP, Boody BS, Hirsch JA, Conger A, Harper K, et al. Pain location and exacerbating activities associated with treatment success following basivertebral nerve ablation: an aggregated cohort study of multicenter prospective clinical trial data. Pain medicine (Malden, Mass). 2022;23(Suppl 2):S14-S33. doi: 10.1093/pm/pnac069.

58. Nilsson-Wikmar L, Pilo C, Pahlback M, Harms-Ringdahl K. Perceived pain and self-estimated activity limitations in women with back pain post-partum. Physiotherapy research international : the journal for researchers and clinicians in physical therapy. 2003;8(1):23-35. doi: 10.1002/pri.269.

59. O'Neill S, Jensen TS, Kent P. Computerized quantification of pain drawings. Scandinavian journal of pain. 2019;20(1):175-189. doi: 10.1515/sjpain-2019-0082.

60. Ohlund C, Eek C, Palmbald S, Areskoug B, Nachemson A. Quantified pain drawing in subacute low back pain. Validation in a nonselected outpatient industrial sample. Spine. 1996;21(9):1021-1031. doi: 10.1097/00007632-199605010-00005.

61. Ohnmeiss DD. Repeatability of pain drawings in a low back pain population. Spine. 2000;25(8):980-988. doi: 10.1097/00007632-200004150-00014.

62. Ohnmeiss DD, Vanharanta H, Ekholm J. Degree of disc disruption and lower extremity pain. Spine. 1997;22(14):1600-1605. doi: 10.1097/00007632-199707150-00015.

63. Ohrbach R, Sharma S, Fillingim RB, Greenspan JD, Rosen JD, Slade GD. Clinical characteristics of pain among five chronic overlapping pain conditions. Journal of oral & facial pain and headache. 2020;34(Suppl):s29-s42. doi: 10.11607/ofph.2573.

64. Pande KC, Khurjekar K, Kanikdaley V. Correlation of low back pain to a high-intensity zone of the lumbar disc in Indian patients. Journal of orthopaedic surgery (Hong Kong). 2009;17(2):190-193. doi: 10.1177/230949900901700214.

65. Parker H, Wood PL, Main CJ. The use of the pain drawing as a screening measure to predict psychological distress in chronic low back pain. Spine. 1995;20(2):236-243. doi: 10.1097/00007632-199501150-00022.

66. Pfingsten M, Baller M, Liebeck H, Strube J, Hildebrandt J, Schops P. [Psychometric properties of the pain drawing and the Ransford technique in patients with chronic low back pain]. Gutekriterien der qualitativen Bewertung von Schmerzzeichnungen (Ransford-Methode) bei Patienten mit Ruckenschmerzen. 2003;17(5):332-340. doi: 10.1007/s00482-003-0223-0.

67. Potier T, Tims E, Kilbride C, Rantell K. Evaluation of an evidence based quality improvement innovation for patients with musculoskeletal low back pain in an accident and emergency setting. BMJ quality improvement reports. 2015;4(1). doi: 10.1136/bmjquality.u205903.w2411.

68. Prins MR, van der Wurff P, Groen GJ. Chronic low back pain patients with accompanying leg pain: the relationship between pain extent and pain intensity, disability and health status. Journal of back and musculoskeletal rehabilitation. 2013;26(1):55-61. doi: 10.3233/BMR-2012-00350.

69. Provenzano DA, Fanciullo GJ, Jamison RN, McHugo GJ, Baird JC. Computer assessment and diagnostic classification of chronic pain patients. Pain medicine (Malden, Mass). 2007;8 Suppl 3:S167-175. doi: 10.1111/j.1526-4637.2007.00379.x.

70. Ris I, Barbero M, Falla D, Larsen MH, Kraft MN, Sogaard K, Juul-Kristensen B. Pain extent is more strongly associated with disability, psychological factors, and neck muscle function in people with non-traumatic versus traumatic chronic neck pain: a cross sectional study. European journal of physical and rehabilitation medicine. 2019;55(1):71-78. doi: 10.23736/S1973-9087.18.04977-8.

71. Rising DW, Bennett BC, Hursh K, Plesh O. Reports of body pain in a dental student population. Journal of the American Dental Association (1939). 2005;136(1):81-86. doi: 10.14219/jada.archive.2005.0032.

72. Sorensen CJ, Johnson MB, Callaghan JP, George SZ, Van Dillen LR. Validity of a paradigm for low back pain symptom development during prolonged standing. The Clinical journal of pain. 2015;31(7):652-659. doi: 10.1097/AJP.0000000000000148.

73. Southerst D, Stupar M, Cote P, Mior S, Stern P. The reliability of measuring pain distribution and location using body pain diagrams in patients with acute whiplash-associated disorders. Journal of manipulative and physiological therapeutics. 2013;36(7):395-402. doi: 10.1016/j.jmpt.2013.05.023.

74. Spyridonis F, Ghinea G. A pilot study to examine the relationship of 3D pain drawings with objective measures in mobility impaired people suffering from low back-pain. Annual International Conference of the IEEE Engineering in Medicine and Biology Society IEEE Engineering in Medicine and Biology Society Annual International Conference. 2010;2010:3895-3898. doi: 10.1109/IEMBS.2010.5627668.

75. Starzec M, Truszczynska-Baszak A, Tarnowski A, Rongies W. Pregnancy-related pelvic girdle pain in Polish and Norwegian women. Journal of manipulative and physiological therapeutics. 2019;42(2):117-124. doi: 10.1016/j.jmpt.2019.03.002.

76. Sturesson B, Uden G, Uden A. Pain pattern in pregnancy and "catching" of the leg in pregnant women with posterior pelvic pain. Spine. 1997;22(16):1880-1884. doi: 10.1097/00007632-199708150-00013.

77. Sanders NW, Mann NH, 3rd, Spengler DM. Pain drawing scoring is not improved by inclusion of patient-reported pain sensation. Spine. 2006;31(23):2735-2733. doi: 10.1097/01.brs.0000244674.99258.f9.

78. Sandmark H, Nisell R. Measurement of pain among electricians with neck dysfunction. Scandinavian journal of rehabilitation medicine. 1994;26(4):203-209. PMID: 7878395.

79. Sator-Katzenschlager S, Fiala K, Kress HG, Kofler A, Neuhold J, Kloimstein H, et al. Subcutaneous target stimulation (STS) in chronic noncancer pain: a nationwide retrospective study. Pain practice : the official journal of World Institute of Pain. 2010;10(4):279-286. doi: 10.1111/j.1533-2500.2009.00351.x.

80. Serif T, Ghinea G. Recording of time-varying back-pain data: a wireless solution. IEEE transactions on information technology in biomedicine : a publication of the IEEE Engineering in Medicine and Biology Society. 2005;9(3):447-458. doi: 10.1109/titb.2005.847514.

81. Tesarz J, Gerhardt A, Leisner S, Janke S, Treede R-D, Eich W. Distinct quantitative sensory testing profiles in nonspecific chronic back pain subjects with and without psychological trauma. Pain. 2015;156(4):577-586. doi: 10.1097/01.j.pain.0000460350.30707.8d.

82. Vingard E, Mortimer M, Wiktorin C, Pernold R P T G, Fredriksson K, Nemeth G, Alfredsson L. Seeking care for low back pain in the general population: a two-year follow-up study: results from the MUSIC-Norrtalje Study. Spine. 2002;27(19):2159-2165. doi: 10.1097/00007632-200210010-00016.

83. Vogel S, Magerl W, Treede R-D, Schilder A. Dose-dependent pain and pain radiation after chemical stimulation of the thoracolumbar fascia and multifidus muscle: a single-blinded, cross-over study revealing a higher impact of fascia stimulation. Life (Basel, Switzerland). 2022;12(3). doi: 10.3390/life12030340.

84. Vucetic N, Maattanen H, Svensson O. Pain and pathology in lumbar disc hernia. Clinical orthopaedics and related research. 1995 (320):65-72. PMID: 7586844.

85. Wand BM, Keeves J, Bourgoin C, George PJ, Smith AJ, O'Connell NE, Moseley GL. Mislocalization of sensory information in people with chronic low back pain: a preliminary investigation. The Clinical journal of pain. 2013;29(8):737-743. doi: 10.1097/AJP.0b013e318274b320.

86. Tucker KJ, Fels M, Walker SR, Hodges PW. Comparison of location, depth, quality, and intensity of experimentally induced pain in 6 low back muscles. The Clinical journal of pain. 2014;30(9):800-808. doi: 10.1097/AJP.0000000000000026.

87. Turp JC, Kowalski CJ, O'Leary N, Stohler CS. Pain maps from facial pain patients indicate a broad pain geography. Journal of dental research. 1998;77(6):1465-1472. doi: 10.1177/00220345980770061101.

88. Turp JC, Kowalski CJ, Stohler CS. Temporomandibular disorders--pain outside the head and face is rarely acknowledged in the chief complaint. The Journal of prosthetic dentistry. 1997;78(6):592-595. doi: 10.1016/s0022-3913(97)70010-6.

89. Uden A, Astrom M, Bergenudd H. Pain drawings in chronic back pain. Spine. 1988;13(4):389-392. doi: 10.1097/00007632-198804000-00002.

90. van den Hoven LHJ, Gorter KJ, Picavet HSJ. Measuring musculoskeletal pain by questionnaires: the manikin versus written questions. European journal of pain (London, England). 2010;14(3):335-338. doi: 10.1016/j.ejpain.2009.06.002.

91. Wesollek K, Kowark A, Czaplik M, Rossaint R, Kowark P. Pain drawing as a screening tool for anxiety, depression and reduced health-related quality of life in back pain patients: A cohort study. PloS one. 2021;16(10):e0258329. doi: 10.1371/journal.pone.0258329.

92. Bryner P. Extent measurement in localised low-back pain: a comparison of four methods. Pain. 1994;59(2):281-285. doi: 10.1016/0304-3959(94)90081-7.

93. Faucett J, Meyers J, Tejeda D, Janowitz I, Miles J, Kabashima J. An instrument to measure musculoskeletal symptoms among immigrant Hispanic farmworkers: validation in the nursery industry. Journal of agricultural safety and health. 2001;7(3):185-198. doi: 10.13031/2013.5442.

94. Albarran J, Durham B, Gowers J, Dwight J, Chappell G. Is the radiation of chest pain a useful indicator of myocardial infarction? A prospective study of 541 patients. Accident and emergency nursing. 2002;10(1):2-9. doi: 10.1054/aaen.2001.0304.

95. Cooper G, Bailey B, Bogduk N. Cervical zygapophysial joint pain maps. Pain medicine (Malden, Mass). 2007;8(4):344-353. doi: 10.1111/j.1526-4637.2006.00201.x.

96. Louw A, Schmidt SG, Louw C, Puentedura EJ. Moving without moving: immediate management following lumbar spine surgery using a graded motor imagery approach: a case report. Physiotherapy theory and practice. 2015;31(7):509-517. doi: 10.3109/09593985.2015.1060656.

97. Ohnmeiss DD, Vanharanta H, Ekholm J. Relation between pain location and disc pathology: a study of pain drawings and CT/discography. The Clinical journal of pain. 1999;15(3):210-217. doi: 10.1097/00002508-199909000-00008.

98. Ohnmeiss DD, Vanharanta H, Ekholm J. Relationship of pain drawings to invasive tests assessing intervertebral disc pathology. European spine journal : official publication of the European Spine Society, the European Spinal Deformity Society, and the European Section of the Cervical Spine Research Society. 1999;8(2):126-131. doi: 10.1007/s005860050141.

99. Ohnmeiss DD, Vanharanta H, Guyer RD. The association between pain drawings and computed tomographic/discographic pain responses. Spine. 1995;20(6):729-733. doi: 10.1097/00007632-199503150-00015.

100. Waling K, Sundelin G, Ahlgren C, Jarvholm B. Perceived pain before and after three exercise programs--a controlled clinical trial of women with work-related trapezius myalgia. Pain. 2000;85(1-2):201-207. doi: 10.1016/s0304-3959(99)00265-1.

101. Mann NH, 3rd, Brown MD, Hertz DB, Enger I, Tompkins J. Initial-impression diagnosis using low-back pain patient pain drawings. Spine. 1993;18(1):41-53. doi: 10.1097/00007632-199301000-00008.

102. Petersen EJ, Thurmond SM. Differential diagnosis in a patient presenting with both systemic and neuromusculoskeletal pathology: resident's case problem. The Journal of orthopaedic and sports physical therapy. 2018;48(6):496-503. doi: 10.2519/jospt.2018.7652.

103. Sanders NW, Mann NH, 3rd. Automated scoring of patient pain drawings using artificial neural networks: efforts toward a low back pain triage application. Computers in biology and medicine. 2000;30(5):287-298. doi: 10.1016/s0010-4825(00)00013-5.

104. Spyridonis F, Ghinea G. 3-D pain drawings and seating pressure maps: relationships and challenges. IEEE transactions on information technology in biomedicine : a publication of the IEEE Engineering in Medicine and Biology Society. 2011;15(3):409-415. doi: 10.1109/TITB.2011.2107578.

105. Takata K, Hirotani H. Pain drawing in the evaluation of low back pain. International orthopaedics. 1995;19(6):361-366. doi: 10.1007/BF00178350.

106. Toomingas A. Characteristics of pain drawings in the neck-shoulder region among the working population. International archives of occupational and environmental health. 1999;72(2):98-106. doi: 10.1007/s004200050344.

107. Visscher CM, Lobbezoo F, de Boer W, van der Meulen M, Naeije M. Psychological distress in chronic craniomandibular and cervical spinal pain patients. European journal of oral sciences. 2001;109(3):165-171. doi: 10.1034/j.1600-0722.2001.00008.x.

108. Mann NH, 3rd, Brown MD, Enger I. Statistical diagnosis of lumbar spine disorders using computerized patient pain drawings. Computers in biology and medicine. 1991;21(6):383-397. doi: 10.1016/0010-4825(91)90040-g.
