# Supplementary material for: Sex Representation and User Preferences in Pain Drawing Body Charts in Back Pain Research: Multimethod Study
Source: JMIR Hum Factors. 2026 Feb 9;13:e76175. doi: 10.2196/76175 (PMC12885194; doi:10.2196/76175)
Supplement: Multimedia Appendix 4 [file humanfactors-v13-e76175-s004.docx]

## Multimedia Appendix 4

Body charts with 100% agreement among all six experts

| **PD body chart** | **Authors** | 1. **Sex of study participants as reported in article** 2. **Sex of body chart as reported in article** 3. **Sex of body chart as assessed by our experts** |
| --- | --- | --- |
| 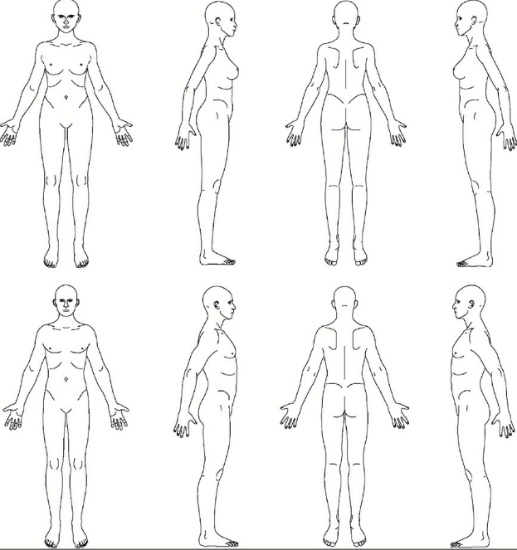 | Barbero, Moresi [1] | 1. Male and female 2. Male and female 3. Male and female |
| 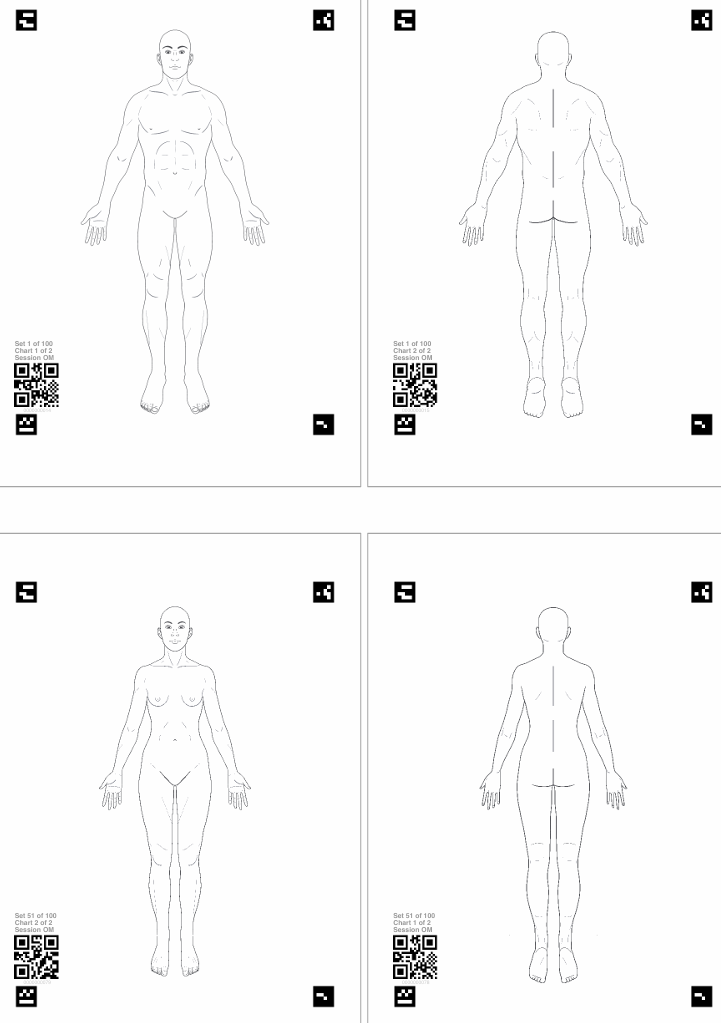 | Barbero, Piff [2] | 1. Male and female 2. Male and female 3. Male and female |
| 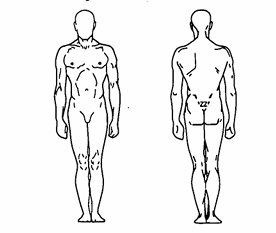 | Chan, Goldman [3] | 1. Male and female 2. Not reported 3. Male |
| 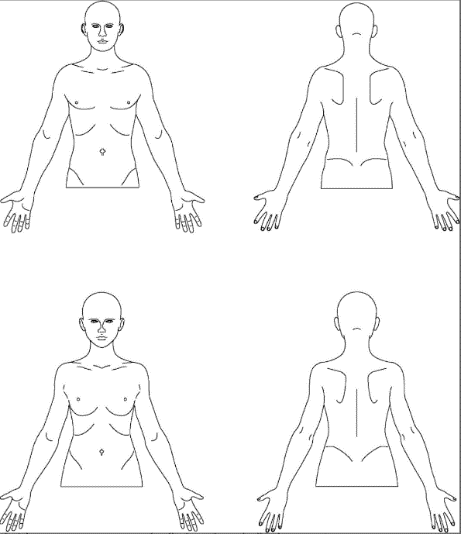 | Cruder, Falla [4] | 1. Male and female 2. Male and female 3. Male and female |
| 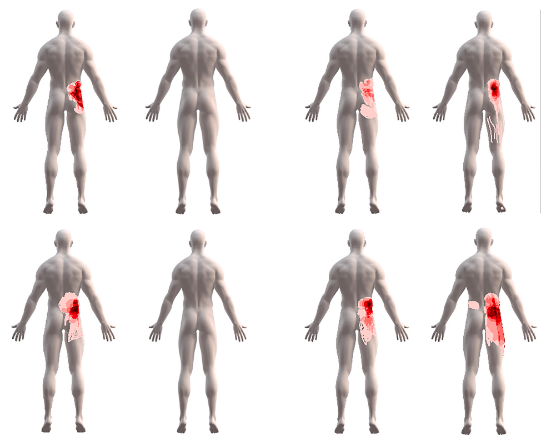 | Galve Villa, Palsson and Boudreau [5] | 1. Male and female 2. Not reported 3. Male |
| 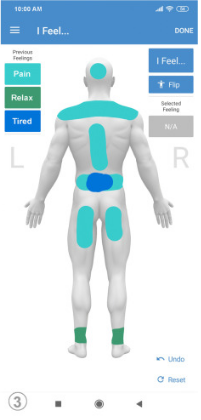 | Goldstein, Ashar [6] | 1. Male and female 2. Not reported 3. Male |
| 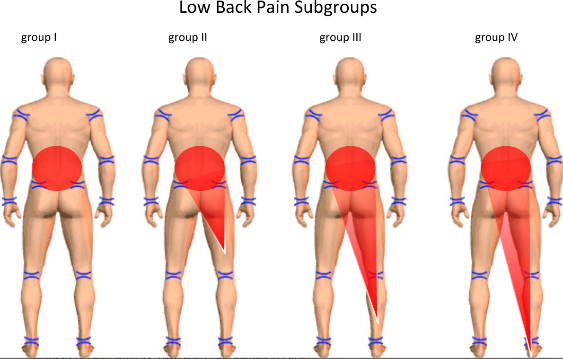 | Hullemann, Keller [7] | 1. Not reported 2. Not reported 3. Male |
| 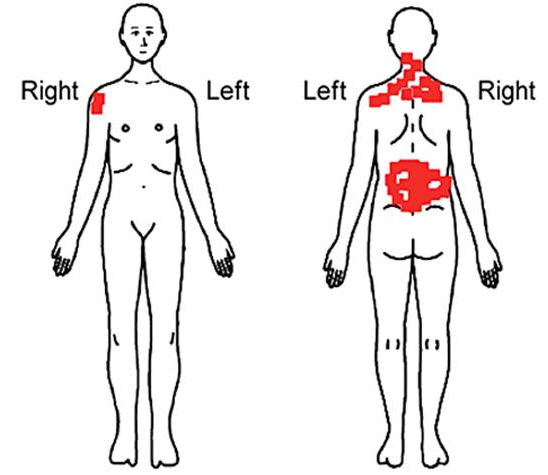 | Provenzano, Fanciullo [8] | 1. Male and female 2. Not reported 3. Androgynous |
| 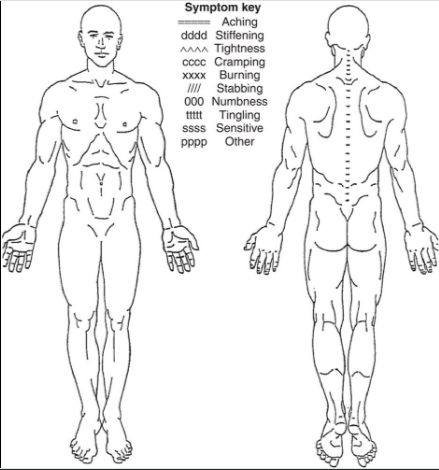 | Sorensen, Johnson [9] | 1. Male and female 2. Not reported 3. Male |
| 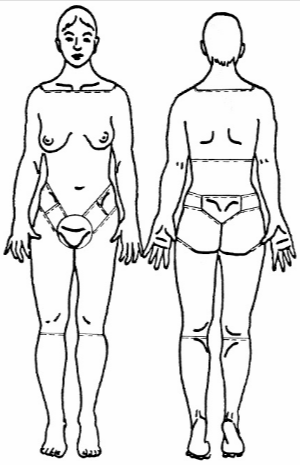 | Sturesson, Uden and Uden [10] | 1. Female 2. Not reported 3. Female |
| 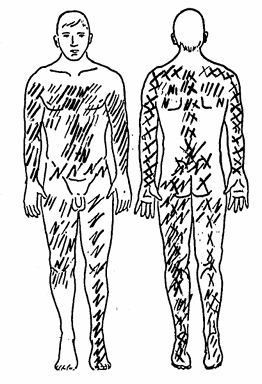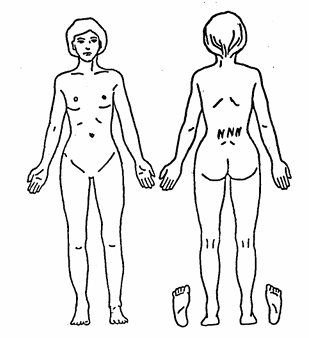 | Uden, Astrom and Bergenudd [11] | 1. Male and female 2. Not reported 3. Male and female |

## References

1. Barbero M, Moresi F, Leoni D, Gatti R, Egloff M, Falla D. Test-retest reliability of pain extent and pain location using a novel method for pain drawing analysis. European journal of pain (London, England). 2015;19(8):1129-1138. doi: 10.1002/ejp.636.

2. Barbero M, Piff M, Evans D, Falla D. Do metrics derived from self-reported and clinician-reported pain drawings agree for individuals with chronic low back pain? Musculoskeletal science & practice. 2023;68:102871. doi: 10.1016/j.msksp.2023.102871.

3. Chan CW, Goldman S, Ilstrup DM, Kunselman AR, O'Neill PI. The pain drawing and Waddell's nonorganic physical signs in chronic low-back pain. Spine. 1993;18(13):1717-1722. doi: 10.1097/00007632-199310000-00001.

4. Cruder C, Falla D, Mangili F, Azzimonti L, Araújo LS, Williamon A, Barbero M. Profiling the location and extent of musicians’ pain using digital pain drawings. Pain Practice. 2018 2018/01/01;18(1):53-66. PMID: 28466572. doi: 10.1111/papr.12581.

5. Galve Villa M, Palsson TS, Boudreau SA. Spatiotemporal patterns of pain distribution and recall accuracy: a dose-response study. Scandinavian journal of pain. 2022;22(1):154-166. doi: 10.1515/sjpain-2021-0032.

6. Goldstein P, Ashar Y, Tesarz J, Kazgan M, Cetin B, Wager TD. Emerging clinical technology: application of machine learning to chronic pain assessments based on emotional body maps. Neurotherapeutics : the journal of the American Society for Experimental NeuroTherapeutics. 2020;17(3):774-783. doi: 10.1007/s13311-020-00886-7.

7. Hullemann P, Keller T, Kabelitz M, Freynhagen R, Tolle T, Baron R. Pain drawings improve subgrouping of low back pain patients. Pain practice : the official journal of World Institute of Pain. 2017;17(3):293-304. doi: 10.1111/papr.12470.

8. Provenzano DA, Fanciullo GJ, Jamison RN, McHugo GJ, Baird JC. Computer assessment and diagnostic classification of chronic pain patients. Pain medicine (Malden, Mass). 2007;8 Suppl 3:S167-175. doi: 10.1111/j.1526-4637.2007.00379.x.

9. Sorensen CJ, Johnson MB, Callaghan JP, George SZ, Van Dillen LR. Validity of a paradigm for low back pain symptom development during prolonged standing. The Clinical journal of pain. 2015;31(7):652-659. doi: 10.1097/AJP.0000000000000148.

10. Sturesson B, Uden G, Uden A. Pain pattern in pregnancy and "catching" of the leg in pregnant women with posterior pelvic pain. Spine. 1997;22(16):1880-1884. doi: 10.1097/00007632-199708150-00013.

11. Uden A, Astrom M, Bergenudd H. Pain drawings in chronic back pain. Spine. 1988;13(4):389-392. doi: 10.1097/00007632-198804000-00002.
